# **First report of** ***Hexametra angusticaecoides*** **Chabaud & Brygoo, 1960 (Nematoda: Ascarididae) in a population of captive central bearded dragons,** ***Pogona vitticeps*** **Ahl (Squamata: Agamidae)**

**DOI:** 10.1007/s11230-024-10202-y

**Published:** 2024-11-19

**Authors:** Laura Hernández-Hurtado, Jacinto Gomes, Luisa Pereira, Maria João Vila-Viçosa, Carlos Gutiérrez-Gutiérrez

**Affiliations:** 1 Polytechnic Institute of Portalegre, Biosciences School of Elvas, 7350-092 Elvas, Portugal; 2 VALORIZA—Research Centre for Endogenous Resource Valorization, 7300-555 Portalegre, Portugal; 3 Department of Veterinary Medicine, Victor Caeiro Parasitology Laboratory, School of Science and Technology & MED—Mediterranean Institute for Agriculture, Environment and Development, University of Évora, Pólo da Mitra, Ap. 94, 7006-554 Évora, Portugal; 4 NemaLab/MED—Mediterranean Institute for Agriculture, Environment and Development & CHANGE—Global Change and Sustainability Institute, Instituto de Investigação e Formação Avançada, Universidade de Évora, Pólo da Mitra, Ap. 94, 7006-554 Évora, Portugal

## Abstract

Between 2022 and 2023 ascaridoid nematodes of the genus *Hexametra* were recovered from two captive central bearded dragons (*Pogona vitticeps* Ahl) in the Extremadura region of Spain. These nematodes were identified by an integrative approach combining morpho-anatomical and molecular data. We used two rRNA regions (D2–D3 expansion segments of the 28S, and ITS fragments), and the partial region of the cytochrome oxidase I gene (COI mtDNA) sequences. *Hexametra angusticaecoides* Chabaud & Brygoo, 1960 (Ascarididae) was morphologically characterized and illustrated using light and scanning electron microscopy. Bayesian inference (BI) and Maximum Likelihood (ML) phylogenetic trees for these genetic markers established relationships that highlight the importance of using molecular and phylogenetic data for accurate species identification within the genus *Hexametra*. Phylogenetic trees indicated that the Spanish isolates of *H. angusticaecoides* were clustered in a well-supported clade together with other isolates from Central Europe of the same species. Molecular phylogenetic findings also reflect that there was a higher intraspecific variation for ITS genetic markers at the intraspecific level. Phylogenetic results based on 28S rRNA suggest that Ascarididea was divided into three monophyletic major clades. To our knowledge, this is the first report of this species parasitizing bearded dragons, extending the host range of this species. These findings represent the first formal record of *H*. *angusticaecoides* in Spain.

**Supplementary Information:**

The online version contains supplementary material available at 10.1007/s11230-024-10202-y.

## Introduction

Ascarididae Baird, 1853 is one of the most diverse and biologically versatile families within the phylum Nematoda paraziting animals (Okulewicz et al., 2002). They are one of the largest groups in terms of the number of taxonomically recognized genera (Hodda, 2022) including gastrointestinal parasites of reptiles, amphibians, and other vertebrates. Notably, it includes some clinical and socio-economically important helminth parasites of humans (Gazzinelli et al., 2012). The genus *Hexametra* Travassos, 1920 has seven valid species, subdivided into two groups based on morphological characters and host specificity (Sprent, 1978; Bowman, 1984; Baker, 1987): (i) *Hexametra* species occurring in lizards, such as *H*. *hexametra* (Gedoelst, 1916) Travassos, 1920; *H*. *applanata* Linstow, 1899; *H*. *angusticaecoides* Chabaud & Brygoo, 1960; and *H*. *rotundicauda* (Linstow, 1904) Mozgovoy, 1953, and (ii) *Hexametra* species occurring in snakes, such as *H*. *boddaertii* (Baird, 1860) Kreis, 1944; *H*. *quadricornis* (Wedl, 1861) Kreis, 1944; and *H*. *leidyi* Bowman, 1984. Overall, female *Hexametra* spp. are characterized by six uterine branches, inter-labia absent, and an intestinal caecum that can be present or absent (Sprent, 1978; Baker, 1987). Likewise, the shape and length of the lips in females and the shape and length of the copulatory spicules in males, however, differs between the subgroups (Sprent, 1978). Furthermore, the systematic position of several species of this genus and the genus itself has been questioned (Travassos, 1920; Baylis, 1921, 1936; Kreis, 1944; Yorke & Maspletone, 1926; Skrjabin et al., 1951; Mozgovoy, 1953; Hartwich, 1957, 1974; Yamaguti, 1961; Kutzer & Grünberg, 1965; Araujo, 1969; Sprent, 1978; Baker, 1987; Barton et al., 2020; Hodda, 2022).

To date, there is a limited range of host species known for each species of *Hexametra* (Brygoo, 1963; Chabaud et al., 1962; Caballero, 1968; Sprent, 1978; Bowman, 1984; Bursey et al., 1995; Dias et al., 2005; Pinto et al., 2020; McAllister et al., 2011; Morton & Krysko, 2012; Santoro et al., 2013; Peichoto et al., 2016; Carbajal-Márquez et al., 2018; Stets, 2019). Among *Hexametra* spp., *H*. *angusticaecoides* has been most widely reported in lizards, mainly in the family Chamaeleonidae, such as *Furcifer oustaleti* Mocquard (see McAllister et al., 2011), *F. pardalis* Cuvier (see Stets, 2019; Reitl et al., 2020), *Chamaeleo calyptratus* Duméril & Bibron (see Jacobson, 2007; Rataj et al., 2011) and the family Diplodactylidae, such as *Correlophus ciliatus* Guichenot (see Barton et al., 2020). Despite the fact that four *Hexametra* species have been reported as paraziting lizards (Caballero, 1968; Sprent, 1978; Bowman, 1984; Baker, 1987; McAllister et al., 2011; Stets, 2019; Barton et al., 2020; Reitl et al., 2020), only *H*. *angusticaecoides* has been reported in countries in Central and Eastern Europe (Rataj et al., 2011; Stets, 2019; Reitl et al., 2020).

Integrative taxonomy is still considered the most efficient approach for accurate diagnosis and identification (e.g., Zhu et al. 2000; Tokiwa et al., 2014; González-Solís et al., 2019; Barton et al., 2020; Chen & Li, 2023). Until recently, identifications of *Hexametra* spp. were exclusively based on morphology (Chabaud et al., 1962; Sprent, 1978; Vicente et al., 1993). However, morphological approaches present considerable limitations in the differentiation of closely related species (Barton et al., 2020); the large body size makes handling specimens a challenge, and their high inter-specific variability and intra-specific plasticity in morphological traits make species identification within the genus *Hexametra* a complex and time-consuming task (Barton et al., 2020). Sequencing of RNA-based markers presents a powerful approach for species-level taxonomic identification (e.g., Barton et al., 2020; Reitl et al., 2020; Sharifdini et al., 2021; Barrera et al., 2022; Chen & Li, 2023). Recently, molecular markers based on ribosomal RNA (rRNA) and mitochondrial RNA (mtRNA) have shown a precise and reliable diagnosis at the species level within the family Ascarididae (e.g., Nadler & Hudspeth, 1998; Arizono et al. 2010; Li et al., 2012; Tokiwa et al. 2014; Zhao et al., 2016; Choi et al., 2017; Camp et al., 2018; Fogt-Wyrwas et al., 2019; González-Solís et al., 2019; Gutiérrez-Gutiérrez et al., 2018; Barton et al., 2020; Reitl et al., 2020; Guo et al., 2021; Sharifdini et al., 2021; Barrera et al., 2022; Chen et al., 2022; Zhou et al., 2023; Chen & Li, 2023). However, to date, there are few sequences deposited in the GenBank database, with bonafide species identification for the genus *Hexametra* (see Barton et al. 2020; Reitl et al., 2020), demanding new and updated molecular data. Consequently, the phylogenetic relationships within the Ascarididae are still not clearly revealed (Barton et al., 2020).

*Pogona vitticeps* Ahl, commonly known as central bearded dragons, is an agamid lizard (family Agamidae; order Squamata) native to Central Australia (Grenard, 1999). This reptile is known as one of the most docile lizard species, making them popular exotic pets in close contact with people around the world. Agamid lizard species are commonly infected by a variety of gastrointestinal helminthic parasites (Hallinger et al., 2019). However, there is a general lack of information on the parasites infecting bearded dragons, with no known records of any species from *Hexametra* (Hallinger et al., 2019). Thus, the objectives of this work were: (1) to characterize, describe, and identify specimens of *Hexametra* obtained from captive *P. vitticeps* in Spain; and (2) to ascertain the phylogenetic relationships between isolates of *Hexametra* species and the available sequences of other ascaridid nematode species deposited in Genbank.

## Material and methods

### Nematode collection and brief clinical history of the captive populations of the central bearded dragon

Adult ascaridoid nematodes of the genus *Hexametra* were collected from two captive central bearded dragons (*P. vitticeps*) in July 2022 and September 2023. Both bearded dragons were housed in the herpetological collection at the Environmental Education Center of Extremadura, Badajoz, Spain. These lizards were in captivity at the same time. These reptiles were individually housed in PVC cages (70 × 56 × 40 cm) and were not co-housed with other reptiles. The basking spot during the light photoperiod reached 35–40°C in the lateral part of the terrarium. The photoperiod was set to 12/12 hr light and dark. The substrate used was red clay soil. The diet of the bearded dragons included vegetables, and shop-bought crickets and mealworms, dusted with calcium carbonate, compatible with the species-specific needs.

**Fig. 1:**
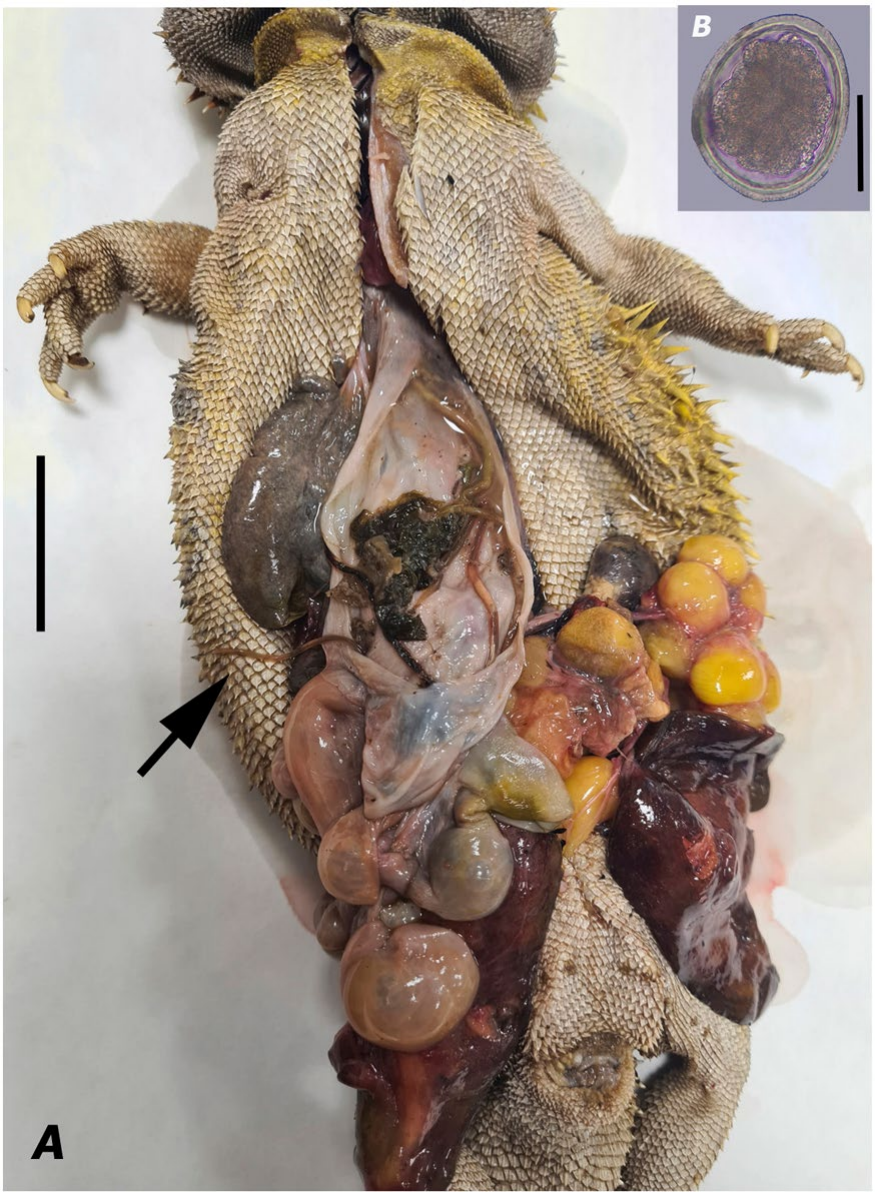
*Pogona vitticeps* Ahl infected with *Hexametra angusticaecoides* Chabaud & Brygoo, 1960. **A** Dissected central bearded dragon: arrow points at the adult specimens of *H. angusticaecoides* within the coelomic cavity; **B** nematode egg. (Scale bars A= 300 mm; B = 42 µm)

Ascaridoid nematodes were recovered from the gastrointestinal tract and fecal samples. Each bearded dragon was housed alone in a different terrarium. Anamnestic data, external examination, and necropsy of the bearded dragon were performed according to Terrell and Stacy (2007). In 2022, one of the infected bearded dragons exhibited a lack of appetite and was consequently moved to the quarantine area for veterinary examination. Throughout the quarantine period, no abnormal signs or symptoms of disease were observed in the specimen. Its behaviour and general activity level remained normal; however, the bearded dragon suddenly died days after being placed in quarantine. Upon examination, there were no signs of bleeding. The external evaluation consisted of an examination of the scales, from the cranium to the caudal area. The head and body were palpated for swellings, wounds, and other abnormalities. The vent was carefully examined to search for accumulation of feces, pathological secretions, ulcerations, prolapse, or foreign bodies. Furthermore, palpation of bone prominences in the animal indicated that it was in very good nutritional condition because the spinal processes in a healthy animal are hard to palpate and not visible; nevertheless, several roundworms were found in the oesophagus and mouth, and nematodes were present throughout the entire coelomic cavity (Fig. 1). The other infected bearded dragon remained asymptomatic, with two observations of fecal samples containing nematode eggs and live adult nematodes during summer ends in 2023 (Fig. 1). These ascaridoids were washed in a physiological saline solution and preserved in 70% alcohol. Voucher specimens were deposited in at the National Museum of Natural Sciences of Madrid, Spain, under accession numbers MNCN-ADN 203875 and MNCN-ADN 203876.

### Morphological studies

After washing in physiological saline solution, fresh ascaridoid specimens were examined with an Olympus® SZX112 microscope and an inverted Leica® DMi1 microscope with an MC170 digital camera; A preliminary morphological identification at the species level using taxonomic keys (Hartwich, 1974; Sprent, 1978; Vicente et al., 1993) was made. Subsequently, specimens were picked into a glass dish for embryos and stored in a physiological saline solution at 4 °C until further processing. For light microscopy (LM) studies, whole or dissected adults (males and females when available) were cleared in lactophenol d`Amann solution for examination as temporary mounts. Light micrographs of specimens mounted on slides were acquired using a light microscope Olympus® BX50 (Hamburg, Germany) with differential interference contrast (DIC) objectives. Phenotypic image analysis and measurements were done using the Olympus DP70 camera Cell® software (Olympus Corp., Tokyo, Japan). All measurements were expressed in micrometers (µm) or milimeters (mm) unless stated otherwise.

For scanning electron microscopy (SEM) studies, fixed specimens were dehydrated in a graded ethanol series, followed by immersion in HMDS (hexamethyldisilazane 98%). After air drying, the specimens were mounted on SEM stubs, sputter-coated with a thin layer of gold (Abdel Rahman, 2021), and observed with a Hitachi S3700N (Tokyo, Japan) SEM coupled to a Bruker (Karlsruhe, Germany) XFlash 5010 SDD Detector system (Gutiérrez-Gutiérrez et al., 2018). The SEM coupled with energy-dispersive X-ray spectrometry (SEM-EDS) experiments were conducted in high vacuum mode with an acceleration voltage of 10 kV. SEM studies were carried out at the Hercules Lab, University of Évora, Portugal.

### DNA extraction, PCR amplification, DNA purification, and sequencing

A tissue slice (subsample of one nematode) of the midbody of adult specimens was excised for molecular study, and the rest of the nematode was used for morphological examination (see section above). Genomic DNA (gDNA) was isolated from an individual female specimen using the NZY Tissue gDNA Isolation Kit (NZYTech, Lisbon, Portugal). The DNA concentration and purity were measured using a NanoDrop-2000C spectrophotometer (ThermoScientific, Wilmington, DE, USA). Subsequently, this gDNA was used to amplify three rDNA fragments: D2–D3 of 28S, the internal transcribed spacer (ITS) 1 and 2 of rRNA and the partial fragment of the COI mtDNA gene (Barton et al., 2020; Cao et al., 2020; Reitl et al., 2020). The PCR was performed in a final volume of 25 μL containing: 1 µL of DNA template, 12.5 µL NZYTaq 2× Green Master Mix (2.5 mM MgCl2, 200 mM dNTPs, 0.2 U/µL DNA Polymerase) (NZYTech, Lisbon, Portugal), 0.6 µL of each primer (10 mM), and 10.3 µL of ddH_2_O. Each rDNA and mtDNA fragment was amplified using a primer pair (Table S1). PCR assays were conducted following the protocols described by Cao et al. (2020) and Barton et al. (2020). PCR cycle conditions included one cycle of 95 °C for 3 min; followed by 30 cycles of 94 °C for 30 s; an annealing temperature of 52 °C (28S-F /28S-R), 53 °C (SS1-F/ SS2-R), and 55 °C (COI-F1/ COI-R2; NC2-F/NC13-R) for 30s, 72 °C for 15–45 s; and one cycle of 72 °C for 7 min. The PCR products were purified as described by Gutiérrez-Gutiérrez et al. (2020) and were used as a template for direct sequencing on a DNA multicapillary sequencer ABI 3730xl DNA Sequencer (Applied Biosystems, Foster City, CA, USA), using a BigDye Terminator V3.1 Cycle Sequencing Kit at the STABVIDA facilities (Caparica, Portugal). The newly obtained D2-D3 of 28S, ITS regions, and COI sequences were deposited in the GenBank and compared with those of other nematode sequences available in the GenBank database using the BLAST homology search program (Altschul et al., 1990).

### Molecular phylogenetic analyses

The newly obtained rRNA sequences (D2–D3 expansion segments of the 28S rRNA gene) and mtRNA sequences (partial COI mtRNA gene) from the *Hexametra* species found in this survey, together with other available sequences of other closely related ascaridoid species, including members of family Ascarididae, from the National Center for Biotechnology Information (NCBI) were used for phylogenetic analyses (Table 1). Outgroup taxa for each phylogenetic tree was chosen belonging to the infraorder Ascaridomorpha, but also residing outside the family Ascarididae. MAFFT v. 7 were used for aligning of the sequences obtained with default parameters (Katoh et al., 2019). Sequence alignments were visualized with ClustalX2 (Larkin et al., 2007) and edited by Gblocks v. 0.91b (Castresana, 2000) with less stringent selection Gblocks parameters (www.phylogeny.fr, Phylogeny.fr platform, accessed on 13 August 2024).

**Table 1:** GenBank accession numbers of DNA sequences of various nematode taxa that were used to construct 28S, ITS1, ITS2 and COI phylogenetic trees in this study

Taxa name	Family	GenBank accession number				References
		28S	ITS1	ITS2	COI	
*Angusticaecum holopterum*	Ascarididae	–	–	–	FM178546	Unpublished
*Anisakis simplex*	Anisakidae	–	–	–	GQ132133	Noguera et al. (2009)
*Ascaris lumbricoides*	Ascarididae	U94751	–	–	–	Nadler and Hudspeth (1998)
*Ascaris suum*	Ascarididae	U94752	–	–	–	Nadler and Hudspeth (1998)
*Baylisascaris ailuri*	Ascarididae	MG937778	–	–	–	Camp et al. (2018)
*Baylisascaris columnaris*	Ascarididae	MG937773	–	–	KY580736	Camp et al. (2018); Choi et al. (2017)
*Baylisascaris devosi*	Ascarididae	MG937776; MN960313	–	–	–	Camp et al. (2018); Sharifdini et al. (2021)
*Baylisascaris laevis*	Ascarididae	ON994376	–	–	–	Barrera et al. (2022)
*Baylisascaris procyonis*	Ascarididae	MG937775	–	AJ007458	MH795149, MH795150	Camp et al. (2018); Zhu et al. (2000)
*Baylisascaris procyonis*	Ascarididae	–	–	–	MW385465	Unpublished
*Baylisascaris potosis*	Ascarididae	AB893608	–	–	–	Tokiwa et al. (2014)
*Baylisascaris schroederi*	Ascarididae	MG937777	–	–	KJ587806; KJ587825	Camp et al. (2018); Xie et al. (2014)
*Baylisascaris tasmaniensis*	Ascarididae	MG937781	–	–	–	Camp et al. (2018)
*Baylisascaris transfuga*	Ascarididae	MG937780	AB571304	–	–	Camp et al. (2018); Arizono et al. (2010)
*Contracaecum* sp.	Anisakidae	–	–	–	KJ561691	Delgado and García (2015)
*Heterakis spumosa*	Heterakidae	MH571869	–	–	–	Zaleśny et al. (2010)
*Hexametra angusticaocoides*	Ascarididae	OR763391- OR763393; MW386882	OR763797- OR763798; MN876031- MN876035; MW442159	OR763814- OR763815; MN876031- MN876035; MW442159	OR742811- OR742812; MW387515	This study; Reitl et al. (2020); Barton et al. (2020)
*Iheringascaris inquies*	Anisakidae	–	–	–	EU741046*	Jaiswal et al. (2016)
*Lagochilascaris minor*	Ascarididae	–	–	–	MH571130, MH571139	Gutiérrez-Solis et al. (2019)
*Lagochilascaris minor*	Ascarididae	–	–	–	OQ437992-OQ437993	Unpublished
*Ophidascaris baylisi*	Anisakidae	–	–	MW837142	MZ310709	Unpublished
*Ophidascaris robertsi*	Anisakidae.	–	AJ007457	–	–	Zhu et al. (2000)
*Ortleppascaris sinensis*	Ascarididae	–	–	–	KM891741	Zhao et al. (2016)
*Parascaris* sp.	Ascarididae	–	–	–	OQ517637, OQ628067, OP745988-OP745991	Zhou et al. (2023)
*Parascaris* sp.	Ascarididae	–	–	–	MK209667*	Unpublished
*Parascaris equorum*	Ascarididae	MG937783	–	–	MH795158	Camp et al. (2018)
*Parascaris univalens*	Ascarididae	–	–	–	OP745979	Zhou et al. (2023)
*Parascaris univalens*	Ascarididae	–	–	–	MK209651*	Unpublished
*Porrocaecum angusticolle*	Ascarididae	MW441215	–	–	–	Guo et al. (2021)
*Seuratascaris numidica*	Ascarididae	OP348903	–	MG434689-MG434690	–	Chen and Li (2023)
*Seuratascaris physalis*	Ascarididae	OP348909	–	–	–	Chen and Li (2023)
*Seuratascaris schmackeri*	Ascarididae	–	–	–	MN120313	Liu et al. (2022)
*Toxascaris leonina*	Ascarididae	JN256999	–	–	MH937707, KX963448	Li et al. (2012); Fogt-Wyrwas et al. (2019)
*Toxascaris leonina*	Ascarididae	–	–	–	PP434590	Unpublished

(–) Not obtained

*Wrongly identified sequence in Genbank database (see Zhou et al. 2023)

Homogeneities of base frequencies and optional substitution models for 28S rRNA, ITS1 and 2 regions rRNA and COI mtRNA datasets were tested with Kakusan4 (Tanabe, 2011). The base composition homogeneity test before the model selection was significantly homogeneous. Bayesian inference (BI) analysis was performed using the software MrBayes v. 3.2.1 (Ronquist et al., 2012). For BI, the substitution model was selected by the Bayesian information criterion (BIC) and the best‐fit model for each gene was selected, namely the K80 model with gamma-shaped distribution for the 28S rRNA gene and ITS2 fragment of rRNA gene, the K80 model homogeneous for ITS1 fragment of rRNA gene, and the HKY85 model with a gamma-shaped distribution for the COI mtRNA gene. Convergence of the MCMC chain and burn-in length were assessed and checked in Tracer 1.7.1 (Rambaut et al., 2018). A total of 1,000,000 generations were performed for each BI analysis, sampling every 100th tree and discarding the 25% of samples as ‘burn-in’. Furthermore, a maximum likelihood (ML) tree based on COI mtDNA sequences was reconstructed with MEGA7 (Tamura et al., 2011) and under the TN93+G+I model with 10,000 bootstrap (BS) replications. Finally, the final consensus trees were partially edited and visualized using FigTree v. 1.4.3 (Rambaut, 2009).

## Results

### Morphological features and morphometric measurements

**Fig. 2:**
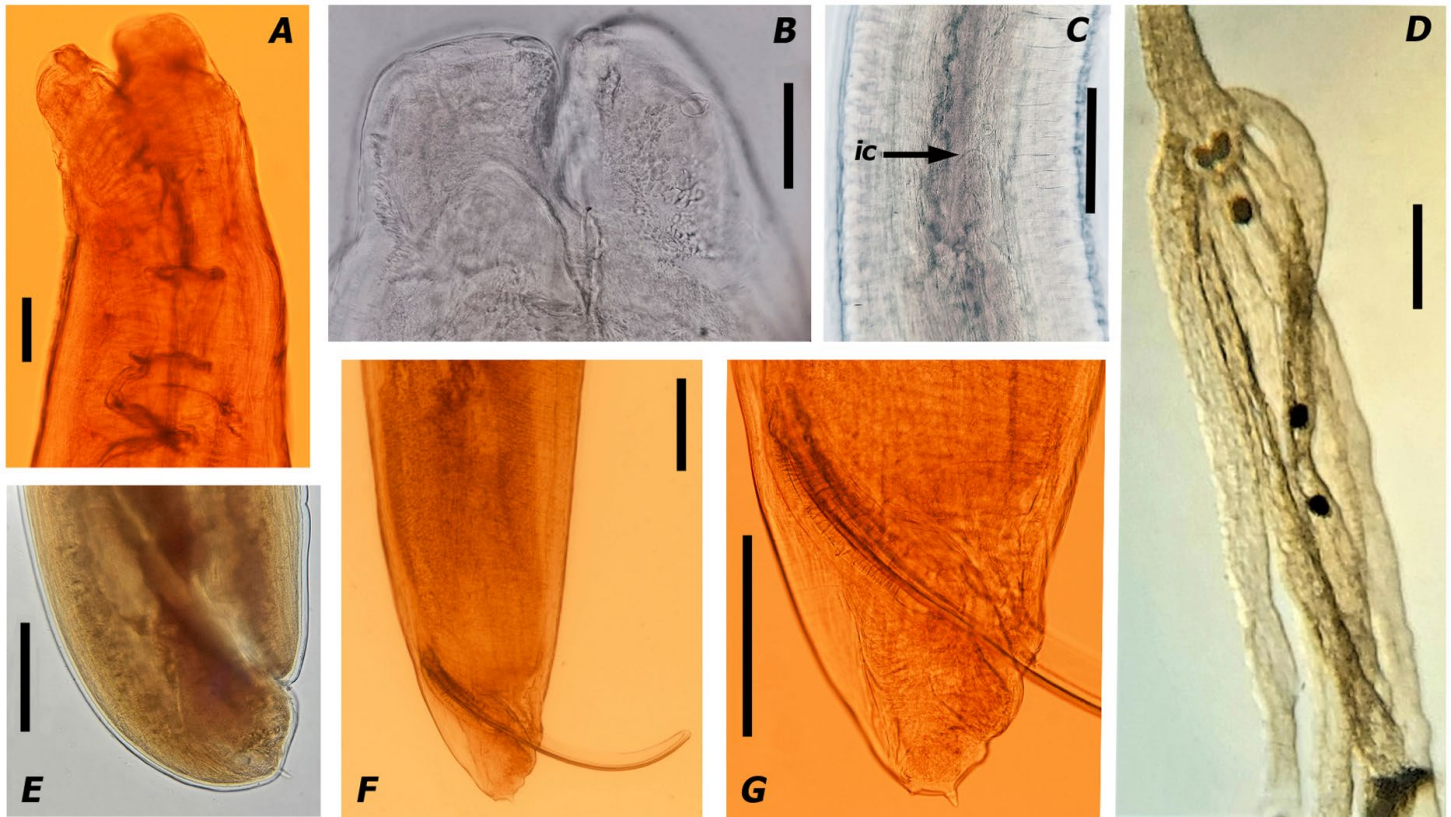
Light microscopy micrographs of Hexametra angusticaecoides Chabaud & Brygoo, 1960. A Female labial region; **B** Male labial region; **C** female mid-body region: arrow points at the intestinal caecum; **D** Dissected uteri with six uterine branches in a mature female; **E** Female tail; **F** and **G** Male tail showing the mucron and spicules (Scale bars **A** = 140 μm; B= 50 μm; C= 400 μm; D= 340 μm; E-G= 360μm). (ic intestinal caecum)

**Fig. 3:**
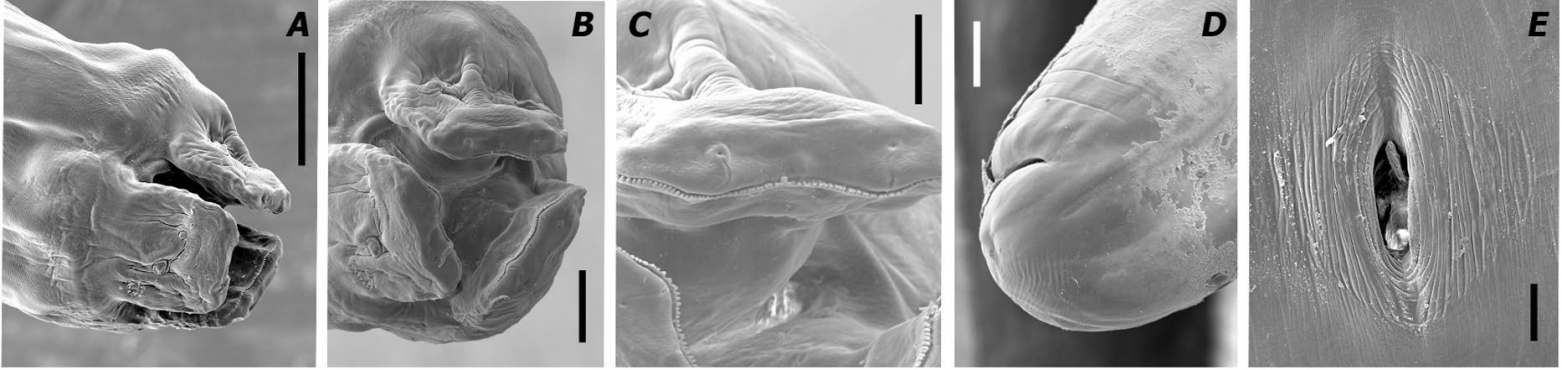
Scanning electron microscopy micrographs of Hexametra angusticaecoides Chabaud & Brygoo 1960. A female anterior region; **B** and **C** the labia in a female; **D** Female tail **E** Vulva (Scale bars A= 140 µm; B, E= 50 µm; C= 25µm; D= 200µm)

Morphological features: Figures 2-3.

Morphometric data: Table 2.

**Table 2:** Measurements of female and male specimens of *Hexametra angusticaecoides* Chabaud & Brygoo, 1960 collected from the bearded dragon, *Pogona vitticeps* Ahl, in the present study and comparative measurements obtained from the literature

Character	*Hexametra angusticaecoides*					
Reference	The present study		Barton et al. (2020)		Chabaud & Brygoo (1960)*	Sprent (1978)
Sex	Male	Female	Male	Female	Male	Males
Host	*Pogona vitticeps*		*Correlophus ciliatus*		*Furcifer oustaleti*	*Chamaeleo* spp.
Body length (mm)	60–72	54–100	39–60	22–69	48	54–95
Max body width (µm)	874–976	990–1320	1080–1380	650–1700	700	830–1500
Vulva from the anterior end (mm)	–	30–42	–	16.13–17.75	–	–
Number of uterine branches		6	–	5 –6	–	–
Subventral lip length (µm)	108–122	119–130	130	65–150	190	100–140
Oesophagus length (mm)	2.40–2.57	3.29–3.44	2.35–3.75	1.75–3.70	3.2	3.5–3.9
Spicule length (µm)	961–1130	–	1000–1100	–	850	820–1100
Nerve ring from anterior extremity (µm)	–	416	480–700	375–800	800	490–800
Excretory pore from anterior extremity (µm)	–	878	880	450–950	920	610–950
Tail length (excluding mucron) (µm)	158–215	359–416	200–220	100–700	230	210–240
Pairs of pre-cloacal papilla	51–68 pairs	–	70–84 pairs	–	57 pairs +1 odd precloacal	40–60 pairs

All measurements in micrometres unless otherwise indicated

(–) Not obtained

*Measurements as cited inBarton et al. (2020)

The descriptions of adult specimens were based on both males and females (Figs. 2 and 3; Table 2). Females containing six uterine branches were characterized morphologically (Fig.2; Table 2). All specimens were morphologically identified as belonging to *H*. *angusticaecoides*.

Brief description: Small to medium body size, usually 60–72 and 54–100 mm long in males and females, respectively; body tapering gradually anteriorly and posteriorly; anterior region with three large lips, usually a larger dorsal lip and two smaller subventral lips; lips wider than long, with a shallow oral groove; inter-labia and postlabial groove absent; dentigerous ridge bears numerous small denticles all-around outline of lips; lips with papillae-like structures; dorsal lip with two papillae; each subventral lip with one papilla and one amphid; alae present in middle oesophageal region; excretory pore located slightly posterior to the nerve ring; oesophagus cylindrical, slightly wider at the oesophagus–intestinal junction; the outline of the intestinal caecum is usually visible; tail short with mucron in older and younger adults; tails of males tend to have a mucron longer than females; tail, usually 359–416 µm long, dorsally convex with bluntly rounded terminus in females; tail, usually 158–215 µm long, dorsally convex-conoid and ventrally bent with 51-68 pairs of precloacal papillae in males; spicules equally well developed and alated; uterus divided into six branches (three females were dissected to determine the number of uterine branches). The eggs were characterized by an 84 µm maximum diameter (Fig. 1).

Remarks: The specimens collected in this study were identified as *H*. *angusticaecoides*, due to the host (lizard) and morphological characteristics of six uterine branches, inter-labia absent, and an intestinal caecum (Mozgovoy, 1953; Hartwich et al., 1974; Sprent, 1978). Both of the bearded dragons in this study were infected with high intensity (6–8 individuals/animal). The morpho-anatomical characters and morphometrical traits closely agree with the original description of this species of *Hexametra* (Chabaud & Brygoo, 1960; Chabaud et al., 1962) and subsequent records (Sprent, 1978; Reitl et al., 2020), except for minor intraspecific differences (see Table 2). Minor differences, such as the number of pairs of pre-cloacal papilla in males (51–68 pairs *vs* 70–84 pairs) and uterine branches in females (six uterine branches *vs* five or six uterine branches) were found with specimens collected by Barton et al. (2020). The nematodes collected in this study were almost indistinguishable from *H. hexametra*, *H. applanata* and *H. rotundicauda* (Sprent, 1978). In this study, the identification of our population was apparently supported by DNA molecular markers (see next section); however, there was not any sequence from *Hexanetra* species with exception of *H*. *angusticaecoides* deposited in GenBank. *Hexametra angusticaecoides* was originally described parasitizing the Malagasy giant chameleon (*F. oustaleti*) in Madagascar (Chabaud & Brygoo, 1960) and later was reported parasitizing other lizards, such as crested geckoes (*Correlophus ciliatus*) in Hong Kong (China) (Barton et al., 2020), the panther chameleon (*F. pardalis*) in Czech Republic (Caballero, 1968; Reitl et al., 2020) and Ukraine (Stets, 2019), and other chameleon species (*Chamaeleo* spp.) in Magadascar (Brygoo, 1963; Caballero, 1968; Sprent, 1978; Chabaud & Brygoo, 1960; McAllister et al., 2011; Morton & Krysko, 2012), as well as snakes belonging to the family Boidae and the family Lamprophiidae in Madagascar (Ghadirian, 1968; Morton & Krysko, 2012). These findings represent the first record of this species in Spain, as well as the first record of this species parasitizing bearded dragons.

### Molecular results and phylogenetic relationships of *H. angusticaecoides* and other members of the genus *Hexametra* and the family *Ascarididae*

For nematode species obtained here, the three rRNA genetic markers (the D2–D3 expansion segments of 28S rRNA, ITS1 and ITS2 rRNA regions) and the partial COI mtRNA gene had an approximate size of 800, 600, 500, and 700 bp, respectively. Ribosomal and mitochondrial sequences of *H*. *angusticaecoides* (OR742811–OR742812; OR763391–OR763393; OR763797–OR763798; OR763814-OR763815) matched with sequences previously deposited in GenBank.

**Fig. 4:**
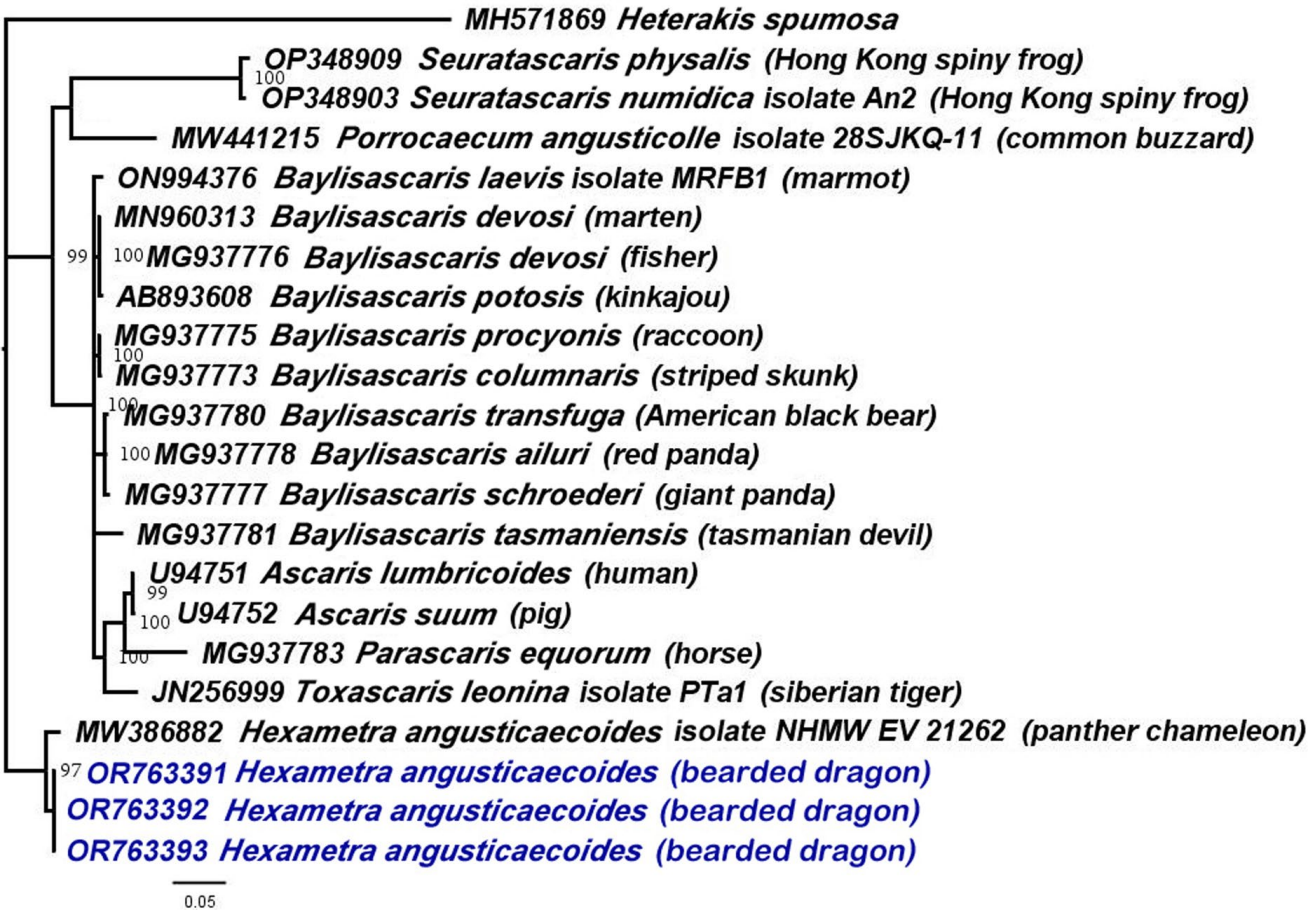
Phylogenetic relationships between *Hexametra angusticaecoides* Chabaud & Brygoo, 1960 and other related members within the family Ascarididae. Bayesian 50% majority rule consensus trees inferred from 28S rRNA sequences alignment (689 bp) under the HKY85 model with a gamma-shaped distribution. Posterior probabilities of more than 0.90 are given for appropriate clades. Newly obtained sequences coloured in navy blue. Scale bar = expected changes per site

The D2–D3 sequences from our population of *H. angusticaecoides* (OR763391–OR763393) matched closely (99–100% similarity) to other sequence of this species in GenBank (MW386882, host: panther chameleon); and the variations among them ranged from 1 to 4 nucleotides and 0 indels. Using Bayesian inference (BI), we inferred the phylogenetic position of *H. angusticaecoides* by using the D2–D3 fragments of 28S rRNA (Fig. 4). The BI tree (50% majority rule consensus tree) of a multiple-edited alignment included 21 sequences of 28S rRNA fragments from the family Ascarididae (Table 1 and Fig. 4) and one outgroup species [*Heterakis spumosa* Schneider, 1866 (Heterakidae),] (Fig. 4). A total of three new sequences were obtained for this ribosomal molecular marker and added to this analysis. The BI tree inferred from the analysis of the 28S sequence alignment contained highly or moderately supported major clades (Fig. 4).

**Fig. 5:**
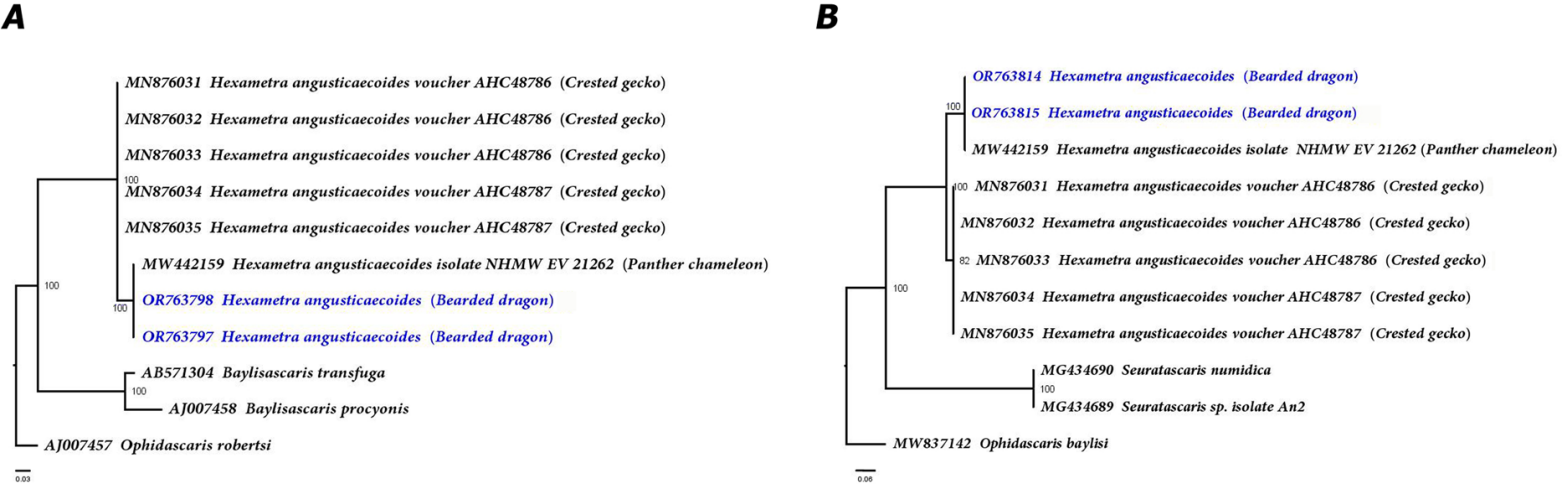
Phylogenetic relationships between *Hexametra angusticaecoides* Chabaud & Brygoo, 1960 and other related members within the family Ascarididae. Bayesian 50% majority rule consensus trees as inferred from ITS1 (**A**) and ITS2 (**B**) sequences alignments (438 bp and 397 bp respectively) under the K80 model homogeneous and K80 model with a gamma-shaped distribution respectively. Posterior probabilities of more than 0.90 are given for appropriate clades. Newly obtained sequences in this study are coloured in navy blue. Scale bar = expected changes per site

The ITS1 segment rRNA gene sequences from *H*. *angusticoicoides* (OR763797– OR763798) matched closely (more than 99% similarity) to a European isolate of the same species deposited in GenBank (MW442159, host: panther chameleon). Our sequences also matched with a Chinese isolate of the same species (93% of similarity) (MN876031, host: crested geckoes); and the variations among these ITS1 sequences ranged from 4 to 38 nucleotides and 3 to 23 indels. Intra-specific variation of ITS1 sequences was detected among the two Spanish populations of *H*. *angusticaecoides* from bearded dragons was of 4 nucleotides (99–100% similarity and 3 indels). Two homogeneous sequences of the ITS2 segment rRNA gene sequences (100% of similarity) for our Spanish population of *H*. *angusticaecoides* (OR763814–OR763815) were similar (90–100% of similarity) to other sequences of this same species deposited in GenBank (MW442159, host: panther chameleon; MN876031, host: crested geckoes). The variations among the ITS2 sequences of these populations of this species were from 0 to 41 nucleotides and 0 to 16 indels. Using Bayesian inference (BI), we inferred the phylogenetic position of *H. angusticaecoides* based on the intergenic transcribed spacer regions ITS1 (Fig. 5A) and ITS2 (Fig. 5B). For each intergenic region, the BI tree (50% majority rule consensus tree) of a multiple-edited alignment included 10 sequences from the family Ascarididae (Table 1 and Fig. 5) and one outgroup species (Fig. 5). A total of four new sequences (two for ITS1 and two for ITS2) were obtained for these two genetic markers and added to this analysis. For each ITS fragment, the BI tree inferred from the analysis of the sequence alignments contained highly supported major clades (Fig. 5). Therefore, the partial ITS1 and ITS2 rRNA regions showed the highest sequence homology with a European isolate of *H*. *angusticaecoides*. Furthermore, we observed higher levels of genetic variation in the ITS1 and ITS2 regions (7% and 10% respectively), between our specimens and the samples from Hong Kong, provided by Barton et al. (2020). However, these findings could reflect higher intraspecific variation for this molecular marker at the intraspecific level. Alternatively, it is possible that there are population level differences between *H*. *angusticaecoides* in Europe and Asia or that the specimens collected in Asia were misidentified.

**Fig. 6:**
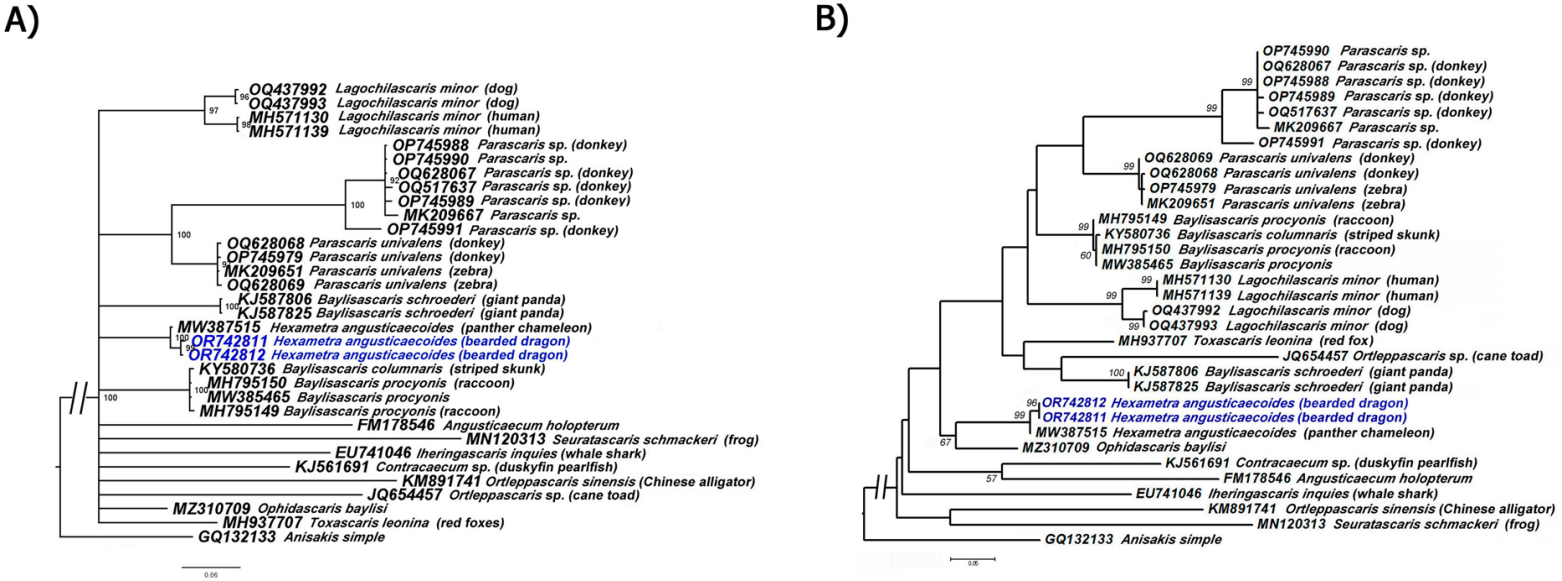
Phylogenetic relationships between *Hexametra angusticaecoides* Chabaud & Brygoo, 1960 and other closely related ascaridoid species including members of family Ascarididae*.* **A** Bayesian 50% majority rule consensus trees as inferred from COI mtRNA sequences alignments (427 bp) under the HKY85 model with a gamma-shaped distribution. Bayesian posterior probabilities Posterior probabilities of more than 0.90 are given for appropriate clades. **B** Maximum likelihood phylogenetic trees (50% majority rule consensus) as inferred from COI mtRNA sequences alignments (427 bp) under the TN93 +G+I model. Numbers indicate the bootstrap values >50. Newly obtained sequences in this study are coloured in navy blue. Scale bar = expected changes per site

Two COI mtRNA gene sequences of *H*. *angusticaecoides* from Spain (OR742811–OR742812) were sequenced and showed a sequence similarity greater than 99%, with minor intra-specific variations (2 nucleotides). Likewise, our COI sequences (OR742811–OR742812) had 99% similarity to those deposited in GenBank for this same species (MW387515, host: panther chameleon); and the variations among these COI sequences varied from 5 to 6 nucleotides. Using Bayesian inference (BI) and Maximum Likelihood (ML), we inferred the phylogenetic position of *H. angusticaecoides* by using the partial COI mtRNA gene sequences (Fig. 6). For the BI tree of this mitochondrial marker, the majority-rule consensus tree consisted of those clades that occurred more than 50% of the time in the collection of trees. The BI and ML tree of the COI mtRNA gene (Fig. 6) was based on a multiple-edited alignment that included 32 COI sequences of ascaridiod nematode species, members of the infraorder Ascaridiomorpha, mostly of the family Ascarididae (29 sequences) and one outgroup species [*Anisakis simplex* (Rudolphi, 1809) Dujardin, 1845 (GQ132133)] (Fig. 6). A total of two new sequences were obtained for this mitochondrial molecular marker and included in this analysis. The BI and ML trees inferred from the analysis of COI sequence alignment showed that a major clade well supported containing mostly highly or moderately supported major clades with quite similar topology with BI trees of D2–D3 segments of the 28S gene.

The generated phylogenetic tree inferred from 28S rRNA and COI mtRNA gene sequences showed congruences between the pattern of branching in the phylogenetic tree and the current taxonomy within the family Ascarididae (Figs. 4 and 6). Likewise, our phylogenetic results based on 28S rRNA divided the Ascarididea into three monophyletic major clades. Furthermore, the generated D2–D3 and COI phylogenetic trees showed a congruent position for *H. angusticaecoides* (Figs. 4 and 6). D2–D3 and COI phylogenetic trees cohesively supported the clustering of our sequences of *H. angusticaecoides* with other isolate of the same species, highlighting these two *H. angusticaecoides* populations as a clearly separated cluster from other morphologically related species, like *Baylisascaris transfuga* Rudolphi, 1819, *Ascaris suum* Goeze, 1782, *A*. *lumbricoides* Linnaeus, 1758 and *Parascaris equorum* Goeze, 1782 (Figs. 4 and 6).

## Discussion

Our work has found the first record of a species of the genus *Hexametra* Travassos, 1920 from the central bearded dragon (*P. vitticeps*). Despite several reports of *Hexametra* spp. in Central and Eastern Europe (Rataj et al., 2011; Stets, 2019; Reitl et al., 2020), this is the first record from Spain.

The majority of captive-bred bearded dragons today are thought to have originated from stock illegally exported from Australia between 1970 and 1990 (Grenard, 1999; Stahl, 1999). Since then, the central bearded dragon (*P. vitticeps*) has been bred in captivity outside Australia, including Europe and USA (Grenard, 1999; Stahl, 1999). In fact, nowadays, this species is one of the most popular reptile pets, with the largest reptile consumer markets present in Europe and USA (Stahl, 1999). Therefore, given that the host is not native to Spain and was in captivity, so it is possible that the bearded dragons obtained the infection from the original captive-bred colony or pet shop from which they were originally acquired. However, this possibility would tend to be refuted as only individual bearded dragon hosted within same terrarium. Barton et al. (2020) did list insects as one possible route, but they suggested previously infected hosts in the same aquarium (with poor cleaning and disinfection between) were the more likely cause of infection in their host lizards. If the bearded dragons in this case were placed into well cleaned aquaria without a history of prior infected animals, then this does tend to suggest the insects could be the cause of infection.

Sprent (1978) proposed that an intestinal caecum can be present or absent; it was present in the current specimens as well as the last records of this species (Barton et al. 2020; Reitl et al., 2020). The morphometric measurements closely agree with the majority of the records of *H. angusticaecoides* (Chabaud & Brygoo, 1960; Chabaud et al., 1962; Sprent, 1978; Reitl et al., 2020), except for minor intraspecific differences, which may be caused by intraspecific variability. However, most of the measurements of the present specimens matched better with the redefinition of this species proposed by Sprent (1978) than the later proposal of Barton et al. (2020) (see Table 2). Despite the fact that most of current measurements fell within the range provided by Barton et al. (2020), the number of pairs of pre-cloacal papilla in males (51–68 pairs vs 70–84 pairs) and of uterine branches in females (six uterine branches vs five or six uterine branches) was different. Barton et al. (2020) suggested that there may be a higher level of morphological variation in the genus in contrast with past literature. However, regarding some of the diagnostic characters, the specimens described by Barton et al. (2020) were rather similar to *H*. *quadricornis*. Likewise, Sprent (1978) denoted that immature specimens of *H. angusticaecoides* are almost morphologically indistinguishable from *H*. *quadricornis*, although this author believed that this last species was unable to develop in lizards. Therefore, the morphological differences among the different diagnoses of this species (Table 2), together with the high prevalence of juveniles and immature adults and the lack of eggs in the fecal samples detected by Barton et al. (2020) make one suspicious about the correct identification. However, the lack of sequences for *H. angusticaecoides* and *H. quadricornis* for comparison (Barton et al., 2020) may have hindered a correct identification.

The present nematodes of *H. angusticaecoides* were molecularly characterized using two rRNA regions (D2–D3 expansion segments of the 28S, and ITS fragments) and a partial region of the cytochrome oxidase I gene (COI mtDNA) sequences. For all these studied molecular markers, our populations of *H. angusticaecoides* emerged as identical to an isolate of the same species retrieved from Central Europe (Reitl et al., 2020). The high degree of genetic variation observed in the ITS regions between the current specimens and samples from China (Barton et al. (2020) suggesting either a potentially higher intraspecific variation for this marker or different species.

In accordance with other systematic studies on Ascarididae (e.g., Choi et al., 2017; Camp et al., 2018; Zhou et al., 2023), our phylogenetic results suggest that the partial COI mtRNA was the most useful molecular marker for species-level identification. In our ML phylogenetic trees, representative sequences for some species occupied different positions. An accurate way to resolve this problem is to sequence voucher specimens. Currently, few populations of *Lagochilascaris minor* Leiper, 1909 and *Baylisascaris* species (e.g., *B*. *columnaris* Leidy, 1856 and *B*. *procyonis* Stefanski & Zarnowski, 1951) have been identified based only on molecular data, occupying distant placements from the sequences of voucher specimens for these same species, and possibly indicating misidentification.

In conclusion, our work updates the biological, morphological and molecular information of *H*. *angusticaecoides* parasitizing captive bearded dragons in Spain. Our study also highlights the value of using rRNA molecular markers for the identification of *Hexametra* spp., when other conventional methods alone based on morphology are inconclusive and time-consuming. Moreover, we generated new molecular markers for precise and unequivocal diagnosis of *H*. *angusticaecoides*, evaluating their genetic diversity and re-establishing molecular phylogenetic relationships that bring new insights into the systematics and the evolution of ascaridiod nematodes. In order to discern among species of *Hexametra*, we urge that more specimens from other members of the genus parasitizing snakes and lizards should be examined following an integrative taxonomy approach.

## Supplementary Information

Below is the link to the electronic supplementary material.

Table S1 List of the primers used in this study
Supplementary file1 (DOCX 16 KB)

## Data Availability

Sequence data that support the findings of this study have been deposited in the Genbank database (OR763391-OR763393; OR763797-OR763798; OR763814-OR763815; OR742811-OR742812)
