# Supplementary material for: First report of Hexametra angusticaecoides Chabaud & Brygoo, 1960 (Nematoda: Ascarididae) in a population of captive central bearded dragons, Pogona vitticeps Ahl (Squamata: Agamidae)
Source: Syst Parasitol. 2024 Nov 19;102(1):6. doi: 10.1007/s11230-024-10202-y (PMC11576818; doi:10.1007/s11230-024-10202-y)
Supplement: Supplementary file 1 — Table S1 List of the primers used in this study Supplementary file1 (DOCX 16 KB) [file 11230_2024_10202_MOESM1_ESM.docx]

**Table S1.** List of the primers used in this study.

| **Primer** | **Sequence (5’-3’)** | **Direction** | **Reference** | **Target (gene)** |
| --- | --- | --- | --- | --- |
| 28S-F | AGCGGAGGAAAAGAAACTAA | forward | Nadler et al. (1998) | 28S |
| 28S-R | ATCCGTGTTTCAAGACGGG | forward | Nadler et al. (1998) | 28S |
| SS1-F | GTTTCCGTAGGTGAACCTGCG | forward | Zhu et al. (2000) | ITS1 |
| SS2-R | AGTGCTCAATGTGTCTGCAA | reverse | Zhu et al. (2000) | ITS1 |
| NC2-F | TTAGTTTCTTTTCCTCCGCT | forward | Zhu et al. (2000) | ITS2 |
| NC13-R | ATCGATGAAGAACGCAGC | reverse | Zhu et al. (2000) | ITS2 |
| COI-F1 | CCTACTATGATTGGTGGTTTTGGTAA TTG | forward | Nunn (1992) | COI |
| COI-R2 | GTAGCAGCAGTAAAATAAGCACG | reverse | Nunn (1992) | COI |
